# Collectanea Medica, Consisting of Anecdotes, Facts, Extracts, Illustrations, Queries, Suggestions, &c.

**Published:** 1818-04

**Authors:** 


### COLLECTANEA MEDICA,

CONSISTING OF

### ANECDOTES, FACTS, EXTRACTS, ILLUSTRATIONS, QUERIES, SUGGESTIONS, &c.

RELATING TO THE

*History or the Art of Medicine, and the Auxiliary Sciences.*

> Quicquid agunt medici,

> nostri farrago libelli.

WE have often expressed a wish that our country readers would direct their attention to subjects of rural economy. No one can be ignorant how much occupied many of those practitioners are who seem fittest for such enquiries. But there are two periods of life in which something may be achieved: when the young physician begins his career, and when the general practitioner finds the necessity of a young partner. At these ages, men, whose professional studies have directed them to physiology, might, we conceive, correct many errors, and even introduce many improvements, in those branches of husbandry which relate to animals raised for the support of man. Under this impression, we have selected the following papers, and shall occasionally add others.

*On the Operation of the Salt-Duties.* From Sir THOMAS BERNARD's Letter to Mr. Vansittart.

As the evil (the want of employment) is not local or temporary, the remedy must not only be general in its effects, but permanent in its duration. Except the greater evil of the renewal of the horrors of war, no measure seems adequate to the object, without the removal of every existing obstacle and impediment to the employment of the labouring classes, so as to augment the call for manual labour, in agriculture, fisheries, and manufactures, and particularly by the cultivation of our waste lands and the extension of our fisheries, to provide new sources of acceptable occupation. Looking to this object, it appears to me, and I shall endeavour to prove, that there is no obstacle or impediment that operates so generally and fatally against the increase of employment for the labouring classes, in these and many other respects, as the existence of the Salt Duties.

There is hardly to be found, in the infinite variety of created matter, any thing more valuable, or more generally applicable to use, than common salt. Composed of two deleterious materials, chlorine and sodium, the united substance is more beneficial and salubrious than it is in the power of our limited understanding to comprehend. Every accession of knowledge discovers new benefits and uses in it. Its spirit is diffused over the boundless ocean. It gives health and purity to the mass of waters, and to the inhabitants of the deep abyss. It preserves every species of food for the use of man, renews the exhausted soil, restores its fertility, and is salubrious and grateful to every kind of animal. In this article of life, England has been peculiarly fortunate. Her brine-springs are rich and abundant; and, what is more extraordinary, are stronger in proportion as they are more and more worked by the pump. A gallon of brine will yield above two pounds and a half of solid salt; whilst the French are not of half the strength of the brine-springs in the county of Chester. Add to this, that the waters which wash the bays and inlets of our coasts are capable of producing an inexhaustible supply of salt; and that the subterraneous treasures of coal, that abound in every part of our island, afford means which no other country possesses, of purifying and crystallizing it for our own use, for the extension of our commerce, and for the supply of the world. No country, indeed, has been in this respect more favoured, except so far as (like the wand of Sancho's physician) the arm of government is extended to prevent our free enjoyment of the bounty of Providence.

In preparing the salt from the brine, there is a refuse part, which is formed by the separation and decomposition of the grosser particles from the pure salt. This is cleared out from the pans, and thrown on the ground, to the amount of several bushels*

* In his Agricultural Survey of Cheshire, p. 238, Mr. Holland notices an experiment made with this refuse salt, where it was at each boiling. Before the excessive increase of the duties (which now are thirty pounds on a ton of salt, the original value of which is about fifteen shillings), the salt proprietors were allowed to dispose of this refuse salt to the farmers, who knew the value of it as a manure, and (however inferior it might be to pure salt) were very glad to purchase as much as they could get of it, at twenty-five shillings a ton; half of which went as a duty to government, and the rest was a clear gain to the salt-proprietor. The late Lord Coventry used to have a regular supply of this manure sent from Droitwich to his place at Crome Court; the excise officer attending to see it moved and laid on the land, and receiving a compliment for his extra attendance. The quantity of this manure which was at one time sold at Northwich alone, to the farmers of that neighbourhood, amounted (as we are informed by Bishop Watson) to near 120,000 bushels annually. This was a very considerable boon to agriculture, and an equal advantage to the salt-proprietor and to government, in respect of the duty it paid. But, when the duties on salt were still more increased, the disposal of this refuse salt was prohibited, to the great regret of the farmers: the country was deprived of the benefit of a cheap and rich manure; and the whole of this refuse salt is now, under a relentless order, carefully swept up by the proprietor's labourers, in the exciseman's presence, and thrown into the river.

In order to promote and encourage the improvement of waste lands, as an additional source of occupation for the disbanded veteran and unemployed labourer, no measure can be proposed so desirable or effectual as the removal of the impediments that arise from the duties on salt; the use of which, in agriculture, is now prohibited by a tax of forty times the value of the article. The supply of means for bringing the soil immediately, and with little expense, into produce, affords the best and most effectual encouragement and incitement to the cultivation of waste and unprofitable lands. It is thus that the introduction of lime into our list of manures has produced, in the course of the last thirty years, the most beneficial and extraordinary effects in this country, by reclaiming millions of acres, hitherto deemed uncultivatable. The vicinity of a lime-quarry, or the power of communicating with one by water-carriage, is marked, in every part of England, by im-

---

spread, in the middle of October, on a piece of sour rushy ground, after the rate of eight bushels to the acre, and, in another part, sixteen bushels. In a short time the vegetation disappeared totally; and, during the month of April following, not a blade of grass was to be seen. In the latter end of the month of May, a most flourishing crop of rich grass made its appearance on that part where the eight bushels had been laid. In the month of July, the other portion produced a still stronger crop. The cattle were remarkably fond of it; and, during the whole ensuing winter, and for several years, the land retained, and yet exhibits, a superior verdure to the neighbouring closes.

proved cultivation and increased fertility: yet lime is not so cheap, nor so powerful, nor so universal, a manure as salt. Lime must be applied in much larger quantities; the carriage is much more expensive, and there are many parts of England where it cannot be had at a price to answer for the husbandman. But salt, duty free, is a great deal cheaper, and (as far as experiments have gone) very superior in power and permanency of effect; and it is to be easily obtained in all the remote and desert parts of the island,—the expense of carriage being comparatively nothing. At the same time, its powerful quality, and the extreme caution required in its application, have occasioned some doubts with regard to its use as a manure. It seems, however, invariably to have answered, when used in the very small quantity of a bushel* to an acre; and, when used in too great abundance, to have been as destructive of vegetation, as it is friendly to it in small and carefully-measured proportions. Bishop Watson accounts for these effects of salt, "when applied as a manure in small quantities, from its efficacy in reducing weeds, dried herbage, dead roots, &c. into a putrid oily mass;" and he goes on to observe, that, when salt is used in a larger proportion, it preserves these matters from corruption, and the fertility of the ground is thereby diminished, or wholly destroyed. This may be confirmed and illustrated by reference to Sir John Pringle's Experiments, which prove that common salt, when used in small quantities, accelerates the putrefaction of animal substances, but, when used in larger quantities, it retards it. Whatever may be the physical cause, it seems now to be practically ascertained, that salt, used in very small quantities, and mixed with loam or mould, is a valuable and powerful manure; but that, in large quantities, it is pernicious. The fertilizing power of a little salt is alluded to in Scripture; where the extraordinary conversions, to be produced by a few illiterate disciples, are compared to the power of a small portion of salt to fertilize an extent of soil:—"Ye are the salt of the earth."

The use of salt as a manure, however, if we were relieved from the duties, would not be confined to waste lands. The practice which existed a few years ago, of applying the refuse salt as a manure, would be renewed, and extended to every part of the kingdom. The farmer would also use the pure and marketable salt, the price of which would not be so much as he formerly paid for refuse salt. Applied in the small quantities I have mentioned, and especially when mixed in the compost dunghill, salt is the best and the cheapest of all manures that can be used in the courses of agriculture. Its value in preserving hay, which has been exposed to wet, has been long known, though the salt duties have now, in a

* I mention one bushel to an acre, on the authority of a gentleman who made a series of experiments on salt as a manure, and held that the proportion of a bushel to an acre answered best, and made the land most productive. Different proportions, howev may suit different soils.

great measure, precluded the use of it. There is a custom in Spain and Portugal, which I have personally witnessed the practice of in North America, of daily placing, on stones, in the sheep-pastures, some dry salt for the use of the sheep. I have seen each of the sheep, in his turn, and with eagerness, take a small portion of it. This is considered as a preservative against the rot, and as contributing to their general health and good condition. It is understood, that a considerable part of the salt which we export, duty free, to America, is used for the purposes of agriculture, though, by the time it reaches the American farmer, it costs him as much as two shillings and sixpence a bushel: and I have no doubt, but, in our humid and uncertain climate, and in the variety of our soils, it would, in small quantities, be found generally useful in preserving our sheep from the rot, and other complaints, hitherto deemed inevitable and incurable. In Spain, the cows are regularly supplied with a little salt; and the increase of their milk, and the benefit which stable-fed cattle derive from it, are confirmed by many authorities. A Cheshire gentleman informs me, that, when he wants extraordinary exertion from his horses, he always gives them a little salt; and this is analogous to the practice in the East, where the camels are allowed salt during the passage of the caravans over the desert to Alexandria, as a support in the extreme fatigue which they undergo.

The advantage of augmenting the number of cultivated acres in this country would certainly be of great use at present, and would have permanent effects on the increase of employment and subsistence in future. In many instances, however, legislative sanctions would be necessary to enable the parties interested to proceed to exclusive cultivation; but, in the millions of acres that surround our coasts, no partition of property, no cultivation of soil, is required: we have only to reap what the bounty of Providence has abundantly supplied. The boundless fields are already white for the harvest; and the labourers are standing all the day idle for want of employment, ready to enter on their task as soon as the financial prohibition is removed. The encouragement of our fisheries has been often in the view of the legislature, but all their measures have been defeated by the salt laws. Bounties, too, have been added, producing little effect, and subject to great abuse; and we have yet to learn, that the effectual and impartial bounty is to leave to the husbandman, the fisherman, the manufacturer, and to all, as far as may be, the free use of that produce which the bounty of Providence has bestowed upon the country. If we really mean that the fisheries should prevail and prosper, as sources of internal supply and external commerce, as providing present employment for our sailors, and future nurseries for our navy; if we desire that the fisherman, and cottager on the sea-coast, should lay in a winter supply of salt-fish for themselves and their neighbours,—we must no longer embarrass them with vexatious conditions, which they cannot understand or comply with, but leave them free to purchase, at a cheap rate, and at the nearest shop, the salt required for curing their fish, and to use it for the benefit of themselves and their country. It has been supposed, that there is already a sufficient allowance of salt for the fisheries, and that it is the fault of those who do not use it: but what sailor, what fisherman, or cottager, can provide what the law requires, proper and secure storehouses for keeping the salt, to be entered with and approved by the excise officer? Who can cure in bulk, or can dry-salt, a hundred weight of fish with only fifty pounds of salt, the present reduced allowance? And, even if the full allowance of salt were restored to the fisherman, and the previous condition of the erection and entry of proper and secure storehouses entirely dispensed with, what uneducated sailor, cottager, or fisherman, could travel through all the different sections of the Acts relating to this subject, the bonds to be given, the annual accounts to be made out, the entries, notices, and permits required, and the penalties and forfeitures in breach of any of the forms prescribed? If an educated lawyer* will do this, he will see the impossibility of

---

* A recent circumstance has occurred in regard to "the Association for the Relief of the Manufacturing and Labouring Poor," where statesmen and lawyers were the acting parties, and every assistance given by government; and yet all their measures baffled by the interference of a petty excise officer. As this is a case which came within my personal observation, I will briefly state the circumstances. In order to provide relief for the poor under the pressure of scarcity, the sum of 17,000*l.* was raised by private subscription in the metropolis. Among other measures adopted, a contract was entered into with the North Sea fishermen, to purchase of them, at the rate of 18*l.* per ton, all the corned cod which they could not otherwise dispose of; and, in the year 1813, six hundred tons of corned cod, and three hundred tons of fresh cod, were supplied and distributed for the maintenance of our own poor, and of the French prisoners then in England. In 1814, the pressure of the scarcity still continuing, the committee resolved to endeavour to double the supply: they, therefore, invested the sum of 2,264*l.* 11s. in the purchase of salt, prepared tanks for curing the fish, and hired double the number of vessels that were employed in the preceding year. When the fishermen were ready to proceed on their voyages, doubts were suggested, and notices given by the excise officer of the district, as to their allowance of salt. An alarm instantly spread among the fishermen; and though, upon the committee's application to the Treasury, an order was obtained for the excise to make the full allowance of salt, duty-free, yet the terror of pains, penalties, and exchequer processes, prevailed among the fishermen, and most of them abandoned their contract for the season. In consequence, the quantity of nine hundred tons, or, rather, (what might have been obtained but for officious interference,) the expected supply of eighteen hundred tons of palatable and nutritious food for the any of the class of labourers and seafaring men, such as I have described, ever taking the benefit of these allowances. But, were the financial fetters of the Salt Laws once removed, we should then see what national industry and national enterprise could effect. Our sea-coasts would swarm with adventurous fishing-boats; new means of employment would be afforded to the necessitous and unemployed; new supplies of life would be conveyed into the interior of the island; new sources of commerce be opened to foreign countries, and nurseries of seamen abound on our sea-coasts. No region on earth is so well adapted to the salt-fish trade. We are a naval power—the ocean is our element. We possess a hardy enterprising race of sailors, now grievously suffering for want of employment; we have shoals of fish surrounding our coasts, and an inexhaustible store of native salt, which may be more easily and much more profitably applied in curing fish than by exportation.

Though manufactures may not supply so great or so desirable an increase of our means of employment as agriculture and the fisheries, yet every addition to our manufactures is a considerable addition to the demand for labour, as well as to the resources of the country: and here, too, the salt duties present the same obstacle to progress and prosperity. On two of our manufactures there is a limited and qualified allowance of the salt duties—on glass, and oxymuriatic acid for bleaching: but there are articles essentially necessary to our manufactures, which the salt duties prevent our making at home; so that we are obliged to purchase from abroad, at much higher prices than we could make them at home. Such are mineral alkali, sal-ammoniac, magnesia, and Glauber salts; which might be made in this country with great advantage, and in great quantities, with a part of the salt which we now export free of duty. In other manufactures, the effect of the salt duties is only to injure the English manufacturer, who, for the salt which his works require, must pay a duty of fifteen shillings a bushel on our own native salt, whilst the foreigner has it duty-free. To enumerate all the uses of salt, in their various processes for facilitating and improving their operations, would require an intimate and confidential knowledge of the secrets of their manufactories; but it does not require that minute degree of information, to be convinced that, on all our manufactures (the exports of which exceeded in value, last year, fifty-three millions), considering them merely as objects of taxation, the revenue suffers more in them only, from the effects of the salt duties, than the salt duties amount to.

Of articles essential to our manufactures, and capable of afford-

---

relief of a suffering population, was reduced to one hundred and fifty tons; being only a twelfth part of what might otherwise have been obtained. The Association was subjected to a heavy loss, and countless loads of fish were lost to the country.

ing an increase of employment for the poor, in case of the repeal, or allowance of the salt-duties, I shall first notice the article of mineral alkali, an ingredient in soap, and in some other of our manufactures. Three tons of salt will make one ton of mineral alkali; the salt, without the duty, costing forty-eight shillings,—and, with the duty, ninety-six pounds. The salt duties, in this instance, are not simply an impediment, but a prohibition; and, in order to obtain this ingredient for soap, &c. our manufacturers have been driven to have recourse to Spain for barilla, the price of which varies very much. During the war, it was as high as eighty pounds per ton, and is as low as thirty pounds at present. If salt were allowed to be used, free of duty, mineral alkali could be made, of a superior quality, and at an inferior price, from our own salt-works. At the same time, the supply of barilla is very insufficient, and our soap-boilers are generally obliged to use other inferior articles instead of it. This year the importation of barilla, though more than usual, is 6500 tons; but that is only a part of what this country requires for soap and other manufactures. It is calculated, that there would be an annual demand at home for 20,000 tons of mineral alkali, and that as much more might be immediately disposed of at foreign markets. Such a regular supply at home would improve the quality of soap, lessen its price, benefit our other manufactures, and afford an advantageous market for 120,000 tons of salt, now sent abroad duty-free. It would set all our salt manufactories to work (many being now shut up), and produce an immediate increase in the demand for labour, not only in the salt-works, and in preparing the new works for making the mineral alkali, and also in making it, but in our other manufactures. It would bear a duty of ten shillings per hundred weight, producing about 400,000*l.* a year to the revenue. Mineral alkali is an article so useful, that it is hardly possible to anticipate the extent of the demand for it, if the salt duties were out of the question; and, with the spirit, science, and capital of our manufacturers, and our inexhaustible supply of salt and coals at less than sixpence a bushel, our advantages in the manufacture of it would be such as to exclude all competition of foreign manufacturers. I am not unaware that, when this has been suggested, the objection on the part of the Excise has been, that, if government were to allow the use of salt, which pays a duty of thirty pounds a ton, for making mineral alkali, to pay only ten pounds a ton, the revenue would lose by it; and that what would be gained by the alkali duty, would be many times over conceded in the salt duty. Now, it does not seem to have occurred, that the salt proposed to be used for making this alkali in England is now exported duty-free, for the benefit of foreign manufacturers; and that, if my sanguine wishes could be realised, and 40,000 tons of mineral alkali annually made from 120,000 tons of salt, which would be otherwise sent abroad duty-free, the revenue would gain 400,000*l.* a year, where it now receives nothing: the country would save nearly as much more of money, now remitted abroad for the purchase of barilla; our manufactures and export trade be augmented; the salt proprietor obtain a better market; the demand for labour be increased; and the public greatly benefited.

Another similar instance occurs in the making of sal ammoniac; an article necessary to our manufactures, and hitherto imported at a considerable expense. This is so useful in working metals, that not less than twenty tons of sal ammoniac are annually used by workers of metals in Birmingham only. Our chemists lately discovered a process for making it from common salt, not only at much less expense, but of a superior quality; but the excise officers soon found out that the salt used in this process was liable to the full duty. Baffled, however, in this, the manufacturers made another effort, by adopting a process for making sal ammoniac from the bittern of common salt-works, which is otherwise waste and refuse: but here again the excise-officers interfered, and decided, that the code of salt laws prohibits this bittern to be converted to any such use, without payment of a duty that amounts to a prohibition. In the consequence, foreigners have made use of our own discovery to establish manufactories of sal ammoniac in France and Germany. In North Britain, however, the use of this bittern is allowed by government, and sal ammoniac is making from it in many parts of Scotland.

During the process of making common salt, it would be easy to separate the magnesia, and thereby greatly to improve the quality of the salt for curing fish and animal food, and at the same time to produce quantities of magnesia for exportation; but the effect of the salt laws is to prohibit the use or sale of magnesia so obtained. As to Glauber salts, the salt duties have had a still more injurious and vexatious operation. I have observed, that there is an allowance of the salt duties for making oxymuriatic acid for bleaching. In preparing this there is a considerable residuum, which had been thrown away. To prevent this waste, a gentleman in Lancashire constructed a large apparatus, consisting of boilers and crystallising vessels, and succeeded in producing from this residuum many tons of very fine Glauber salts, on which he paid to the revenue the then existing duty of thirty pounds a ton. "Government, however," says Mr. Parkes, (from whom I transcribe the fact,) "has forbidden the sale of the residuum; and, consequently, this extensive apparatus has become useless." In these, and other articles of manufacture, though the increase of the demand for labour may not be so considerable as in husbandry and the fisheries, yet if the total amount of what would be added to our manufactures by the repeal of the salt duties be fairly estimated, with its consequences in the increase of commerce, and of the trades and occupations connected with manufactures and commerce, we shall find a considerable and immediate addition would be thereby made to the sources of employment.

It must, indeed, astonish foreigners that an immense prohibitory impost should be laid on an article of British produce, essentially necessary to our manufacturers, agriculturists, and fishermen; whilst Ireland and Scotland are burthened in a lesser degree, and France, and Spain, and all the rest of the world, are entirely free. Very different is this from the policy of ancient governments, recognised by the greatest and highest authority:—"Of whom do the kings of the earth take custom or tribute? of their own children, or of strangers?"—"Of strangers."—"Then are the children free." But with us, and by our laws, the strangers are free, and the children are taxed to forty times the value.

The imposition of a tax so far beyond the real value of the article on which it is imposed, inevitably leads to strictness and severity of language in the regulations respecting it; and, notwithstanding all the pains, penalties, forfeitures, and restrictions, which have been so lavishly bestowed on these acts, payment of the duty is very frequently evaded. Taking into consideration the increase of price charged to the consumer, where an excessive tax has been previously advanced, and that this increase of price is also applied to stolen salt, which has paid no duty at all, there will be little doubt but that the consumer pays twice the amount of what is received on account of this tax into his Majesty's exchequer. This, however, is a minor consideration to that of the pernicious effects which it produces on the moral character of the labouring class. The temptation of forty times the original value on a necessary article of life, becomes so great as to affect their principles, and to convert honest men into scurvy knaves. Thievery, and its concomitant vices, prevail in the neighbourhood of great salt-works to an alarming degree. The salt is of so little value to the proprietor before the duty is paid, and is of so much value to the thief who evades the duty, that this kind of petty plunder is carried on to a considerable extent; so that many farmers in the neighbourhood have a regular supply of salt at very reduced prices, periodically brought to their houses, to cure their bacon, salt their cheeses, and for other domestic purposes. This generates thievish habits among the labouring class, and prepares them for atrocious crimes. Two young men, who were executed a few years ago in Cheshire, for defending their plunder by shooting at an exciseman, and who appeared to be by far the least culpable of their gang, confessed, at the gallows, that petty thefts in salt-works were the origin and cause of their criminal habits, and of the unhappy termination of lives which might otherwise have been a blessing to the community.

---

Another consideration we have often wished to impress on the rural physiologist. It is well known that vegetables are, for the most part, more nutritious in proportion as they abound in saccharine matter, and that this property increases with their ripeness. Now, the process of malting is generally admitted to be nothing more than a higher degree of vegetation preparatory to the development of the infant plant, or, as it is sometimes called, germination. Would it not, then, be good economy to malt all kinds of grain used for fattening, or even for feeding, animals?—

The following paper, by M. Proust, extracted from *Les Annales de Chemie et de Physique*, will illustrate our meaning.

*Analysis of Barley before and after its Germination, and the economical Consequences resulting from it.* By M. Proust.

Barley has always had the character of producing a heavy bread, difficult of digestion, and very inferior to that of wheat or rye: it has even been considered as hurtful to the unhappy country-people whom poverty reduces to the necessity of eating it. It appears that the Romans had not a better opinion of it; for we read in Plutarch, that the generals gave barley instead of wheat to the soldiers who had been cowardly in the day of battle. "As coarse as barley-bread," is also a proverb which shews the low esteem in which this grain has always been held. There is, in short, something in barley which diminishes, or which probably does not participate in, the nourishing qualities of the starch, which it certainly also contains. Analysis alone being capable of determining this question, I began some researches in the year 1802, which, however incomplete, will, I think, be useful towards the knowledge of alimentary substances. They will, without doubt, be found very incomplete, and for the following reason:—there is no part of experimental chemistry in which we are not impeded by difficulties which cannot be surmounted in the first instance, and which are therefore necessarily laid aside for the time. For instance, I think I perceive, in the flour of buck-wheat, that the saccharine matter is deteriorated by a mixture of sour and saline matters, which at first I do not recognise: it is, therefore, necessary to keep this substance for a certain time, either concentrated or dissolved in alcohol, in order that the products it is suspected to contain may assume the forms that shall enable me to ascertain their character. It is the same with numberless other results. Those that I announce, I have seen for the first time. I have not been able to examine them a second time, circumstances having prevented me from resuming the occupation; but I shall be careful to shew the doubts that I entertained respecting each part, in order that they may be again examined by other persons, for the purpose of determining with more certainty than I have been able to do. We will now return to the barley.

*Resin.*—Alcohol extracts from the flour of barley a yellow resinous tincture, which I have also found in the flour of wheat and maize: this resin, when dried, remains pitchy; water has no action on it. It is also to be found again in these grains after they have germinated. I conclude that it forms about the hundredth part of the weight of these different flours.

*Gum and Sugar.*—The flour of barley, washed in cold water, gives an extract that is yellow, sugary, slightly sour, and which alcohol decomposes with facility. Four parts of a sort of gum separate from it, and it holds in solution a fifth, of a honey-like product, the character of which it would be proper to ascertain.

During the evaporation of the washing, it deposits also about three per cent. of shreds, which appear to me to be only gluten; something of this will even remain in the gummy product: the whole, however, in the proportion of about twelve per cent. which is just the quantity that is lost in the washing of wheat-flour. M. Sage has already observed these various results, and he very clearly distinguishes them in his *Analyse des Bleds*, printed in 1776.

*New Product.*—But what may now surprise us is the enormous quantity of an acid powder, coarse or ligneous, which is confounded with the starch in the flour of barley. We have a glimpse of it when we wash a paste of this flour, as if to extract gluten from it, which we do not find; but in its place we meet with something rough and sandy, which is, in fact, the product above-mentioned. To this product, or powder, I had given the Spanish name of *cevadina*, from *cevada*, which signifies barley in that language; but for the French, the word *hordeïne*, from the Latin *hordeum*, designates it better.

This *hordeïne* is not soluble in water: it may be separated from the starch by boiling, for a short time, the eighty-eighth per cent. of residuum which the quintal of barley-flour leaves after washing; and we obtain, by this means, from fifty-four to fifty-six per cent. of a yellow powder, which we should naturally take to be saw-dust, it is so much like it. In order to show more clearly the difference which this powder makes between the flour of barley and that of wheat, I shall here give a table of their composition, not perfectly accurate, but still sufficiently approximative. Thus, the flour of wheat and barley furnish the following proportions to the quintal.

| *Wheat.* | | *Barley.* | |
|---|---|---|---|
| Yellow resin | 1 | Yellow resin | 1 |
| Gummy and sugary extract | 12 | Gummy and sugary extract | 9 |
| Gluten | 12·5 | Gluten | 3 |
| Starch | 74·5 | Starch | 32 |
| | | Hordeïne | 55 |
| | 100 | | 100 |

Thus the starch, which, in cereal grain, is considered as the basis of the aliment which it furnishes, is mixed in barley with a surprising quantity of a dry pulverulent substance, having an appearance completely ligneous. It is, therefore, to the excess of this product, as much as to the want of gluten, that we must attribute the inferiority of barley-bread. Certainly, this *hordeïne*, mixed as it is in barley, with starch, gum, sugar, and gluten, is more nourishing and easy of digestion, than if taken alone, and as a simple aliment; yet, it is also certain, that both mankind and large animals can be nourished, in case of need, with a multitude of vegetable substances, infinitely more compact, inert, and, in appearance, more difficult to soften than *hordeïne*, even if it were indeed pure saw-dust: such as the bark of the pine, the birch, the roots of some ferns, and many other ligneous products, of which, according to travellers, some northern nations make a sort of bread. Finally, this *hordeïne*, whatever it may be, is contained in the bread that is eaten at Paris, although in very small quantity. The analysis of this substance exhibits nothing that distinguishes it from those ligneous vegetables which contain no azote, or scarcely any. By distillation, for instance, there is obtained twenty per cent. of carbonaceous residuum, vinegar, oil, and gases, that retain a part of it, but no appearance of ammonia. After this, yellow bitter matter, which is an indication of a little azote.

*Germination of Barley.*—Here the grain loses weight considerably, for it requires from 145 to 150 grains of germinated barley to make the weight of 100 grains of ordinary barley. As to its component parts, the germination, which is, in fact, only a commencement of vegetation, augments some, diminishes others, and changes considerably the nature of the starch; but a comparative table of the grain before and after germination, will better show the extent of these changes.

| *Ungerminated Barley.* | | *Germinated Barley.* | |
|---|---|---|---|
| Yellow resin | 1 | Resin | 1 |
| Gum | 4 | Gum | 15 |
| Sugar | 5 | Sugar | 15 |
| Gluten | 3 | Gluten | 1 |
| Starch | 32 | Starch | 56 |
| Hordeïne | 55 | Hordeïne | 12 |
| | 100 | | 100 |

In the first place, we must observe, that it takes a much greater quantity of the germinated barley to obtain a quintal of flour from it, than of the ungerminated to obtain the same weight of flour. This circumstance must be kept in mind, when we are comparing, in all the products of the same kind, the augmentation or the diminution that they may have undergone from the effects of the germination. Indeed, we should ascertain what weight of ordinary barley, and of germinated barley, is required in order to obtain a quintal of flour from either. We will now return to the results of the germination.

1. The gum, of which there is not more than four per cent. in the ordinary grain, is from fourteen to fifteen in the germinated. 2. The sugar rises from five to fourteen or fifteen. 3. The gluten is diminished. 4. The starch, of which there is not more than thirty-two per cent. in the ordinary grain, is found (which is most astonishing) to be from fifty-five to fifty-six in the germinated. Now, does this remarkable augmentation proceed from the particular alteration of the starch, of which we shall presently speak? I cannot determine: but, it is certain, that, as we cannot reasonably presume, that the germination produces or creates starch, I

infer that it is very essential that this experiment be repeated, in order to enlighten us as to what is real, and what is illusory, in this singular result. If it be real, it includes a discovery of the greatest interest, namely, the diminution of the *hordeine*, which is infinitely detrimental to the quality of barley-bread, and the augmentation of the starch, which, in a certain degree, brings it nearer to the quality of wheaten-bread. As to the alteration of the starch, which we shall notice presently, the success that attended the experiment, by which very good bread was made from wheat in which the starch had suffered the same change, would justify, in my opinion, the making of germinated barley into bread. Lastly, the *hordeine,* which is diminished from fifty-five to twelve by the germination, what can have become of it?—Is it converted into starch? These are questions which require much research, but we must leave them now, and pursue our present course.

*Alteration of the Starch.*—That the germination is in itself a process which augments the gummy and sugary parts in barley, as in all other grain, is a circumstance already known, not analytically, perhaps, but at least by tasting, which discovers a considerable increase of the sugary product in the sap of all germinated grain. But, I believe, the alteration of the starch has not hitherto been perceived; and I should, perhaps, hesitate to mention it now, if my late examinations of germinated corn had not convinced me that the same alteration is effected in the starch of wheat. In order to judge more positively on this singular alteration, I shall here relate two decisive experiments.

1. Two drams of ordinary barley-flour were dissolved in four ounces of boiling water, having been previously washed cold, in order to separate all gum or sugar, or other extractive matter. The result of this solution, was a paste and a milky-liquid starch, consisting of nothing by which it could be distinguished from the paste of wheat flour.

2. The experiment was repeated with two drams of germinated barley-flour, also previously washed; but here, instead of paste or liquid starch, we obtain a solution which is transparent while it continues warm, slightly milky when cold, which does not coagulate like the liquid starch: on the contrary, it remains liquid; its taste is sweetish, and it does not even acquire consistence by concentration. It is a gummy transparent extract, to which the paste of the flour of ungerminated barley bears no resemblance; but such are also the qualities of the flour of germinated wheat, which, as I have elsewhere observed, yields neither paste nor *bouillie,* although it is always possible to make it into bread. Thus, the solubility acquired by starch, in consequence of the germination, is a particular change which does not at all depend upon the mixture of the gummy products, sugary or extractive, as we might have supposed.

Assuredly, if the germination did not affect the starch with any sort of change, the wort of beer then would be no more than a solution of fifteen per cent. of sugar, of as much gum, and the whole diluted in an enormous quantity of glue or liquid starch, which might furnish fifty-five per cent. of starch. Whereas, on the contrary, the wort is a coloured juice, very liquid, sweet, and especially remarkable for that soft taste slightly perfumed, which is precisely that of beer before it is mixed with hops or yeast.

*Fermentation of Beer.*—This fermentation is not to be compared with that of wine; as, in the latter, the production of alcohol is the principal effect, and that of carbonic acid is but accessary. But, in the fermentation of beer, the case is quite opposite: the formation of carbonic acid in this is the principal product, and there is very little alcohol. A simple experiment will demonstrate this fact:—Place two bottles in the same line, the one filled with beer, and the other with wine; uncork them, and let them remain in this state for twenty-four hours. At the end of that time, the wine, although it will have lost the carbonic acid it may have contained, will retain its alcohol, and will still be wine; the beer, on the contrary, by the dissipation of its carbonic gas, will be greatly injured, and the little alcohol it contains will not prevent it from becoming vapid, indigestible, and destitute of every quality that renders it desirable. Of the three substances that compose beer, namely, gum, modified starch, and sugar, the last is very evidently the principle that causes the fermentation, since the gum and starch are not susceptible of it; but, as there is only a very small quantity of sugar, consequently, there can only be a very weak proportion of alcohol. This is the reason that so little spirit can be extracted from beer by distillation. M. Sage had previously made this observation, and I have also been convinced of it by experiment. Viewed under this aspect, therefore, beer becomes a liquor, capable of certain application by which it may be rendered extremely useful, both in hospitals and to the poor; a few of which I shall point out.

Waters, saturated with carbonic acid, are either medicinal, or they are not: if they are medicinal, their price is always beyond the reach of the poor; however, if a glass of brisk beer, that is, beer containing a considerable proportion of carbonic acid, be mixed with two glasses of fresh water, it furnishes three glasses of acidulated water, which costs so little, that I should think there can be no objection to employing it in hospitals and among the poor; and, would not such a beverage be much preferable to the insipid diet-drinks that are usually given? If barley-water be ordered, why not administer, in preference, that which can be obtained at so little expense, and so speedily, from germinated barley, since it unites all the advantages of the diluting mucilaginous liquids and eminent diuretics? Since, in short, a glass of fresh beer, pure, or weakened with any quantity of water at pleasure, furnishes all that the physician requires in a moistening regimen. It may be said, that the poor can already obtain the simple beverage of barley-water at a small expense; but it is not, in fact, so cheap to them as may be generally supposed, nor is it so medicinal: for instance, a thousand grains, in weight, of chosen barley, which make nearly a handful, after boiling for a length of time in an English pint of water, which was carefully replenished as it wasted, furnished twelve grains of a saline acid extract, soluble in alcohol, and of an earthy salt taste, extremely disagreeable: not even a trace of mucilage. I am not acquainted, either with these salts or the acid, but I preserved them to examine at leisure: what recommendation, therefore, can there be, in a medicinal view, in an atom of saline matter, with nothing sweet, and nothing mucous, to temper its sharpness? But, again, it may be said, that a second decoction will extract from the cracked and swelled grain, principles which the first decoction could not furnish; the second decoction, however, produced but thirty grains of an extract,—a little gummy, it is true, but quite as sour as the first, and this at the expense of much time and a proportion of fuel.

Thus, in hospitals, where a great quantity of diet-drink is daily prepared, the expense of barley is considerable, and is no farther useful, unless it is thought proper to give to poultry. I conclude, from these reflections, that they cannot fail to be desirous, in future, of substituting germinated barley for the ungerminated, unless the empire of custom should prevail over that of reason.

*The Yeast.*—The fermentation expels, at its commencement, all the *hordeïne* that floats, and is intermixed with the wort of the beer: this gives to the yeast the grained, gelatinous, and trembling appearance, that it has when it is sufficiently drained; but a portion of starch is also expelled by the fermentation, and remains in the yeast. When washed cold and dried, the yeast will divide again in cold water like a simple powder, and it is scarcely wetted before a smell of sour starch is perceived, which discovers its nature. Boiling water seems to dissolve it entirely, and the result is a glue not viscous; which is the character of starch, as we have before said, when modified by germination: but a gelatinous powder is then separated, which is the *hordeïne*, that the process of brewing has caused to pass into the wort. If we keep the washed yeast under water, each of these substances resumes its distinguishing character. The starch whitens, and forms into beds, which are neatly separated from the *hordeïne*. The washed and dried yeast always retains a particular smell, and it does not lose the quality of producing fermentation in other substances. I diluted about an ounce of this yeast in a vessel containing four English quarts of water, sugared to twelve degrees of Beaumé's areometer, and the next day it was in a complete state of fermentation; whereas, without this auxiliary, the sweetened water would have produced, after a long time, only a sort of vinegar mixed with a newly-formed gum. The wine it produced was very spirituous, but spoiled by an after-taste of starch very disagreeable. As to the two elements of the yeast, they were at the bottom of the wine, each under their usual aspects of powder of *hordeïne* and starch. Yeast also supplies the place of leaven, as is well known in the composition of bread. To return for a moment to the subject of the change, (which I trust will take place in the mode of using barley in hospitals,) I cannot but think that these researches will induce the medical profession to adopt, without hesitation, a beverage that must be so much more agreeable to the sick, than those which contain only irritating substances, though happily without effect, because they are mostly drowned in a deluge of boiled water.

---

